# Trajectories of Allostatic Load Biomarkers Across the Cognitive Spectrum: Unveiling Patterns in Alzheimer's Disease Risk

**DOI:** 10.1002/alz70856_101642

**Published:** 2025-12-24

**Authors:** Alaa Harb, Juliana Nery Souza‐Talarico, Yelena Perkhounkova, Maria Hein

**Affiliations:** ^1^ University of Iowa, Iowa City, IA, USA; ^2^ University of Iowa, Iowa, IA, USA

## Abstract

**Background:**

Allostatic load (AL), reflecting cumulative biological stress, may contribute to Alzheimer's Disease (AD) risk. Biomarkers of AL may change during progression of cognitively unimpaired (CU) individuals as they develop Mild Cognitive Impairment (MCI) or AD. This study investigates change patterns in AL biomarkers in CU, MCI, and AD individuals.

**Methods:**

Using the Alzheimer's Disease Neuroimaging Initiative (ADNI) database, we analyzed AL changes over 24 months in individuals cognitively unimpaired (CU), MCI and AD. Mean ALI and standard deviations were calculated, and biomarker changes were reported.

**Results:**

At baseline, mean ALI values were similar across groups (CU: 4.3 ± 2.1, MCI: 4.2 ± 2.3, AD: 4.3 ± 2.4, *p* =  0.781). Over 24 months, significant changes in neuroendocrine biomarkers were observed. Cortisol increased most in the MCI group (24, 72.73%), Prolactin in AD (19, 61.29%), and Luteinizing Hormone (LH) and Testosterone in CU (LH: 4, 57.14%; Testosterone: 5, 71.43%).

Inflammatory biomarkers were highest in AD overall (81, 54.73%). Interleukin‐18 (IL‐18) and Tumor Necrosis Factor Alpha (TNF‐α) changes were most frequent in AD (IL‐18: 19, 76.00%; TNF‐α: 20, 76.92%), while IL‐6R increased mainly in MCI (14, 58.33%) and MCP‐3 in AD (17, 58.62%).

**Conclusion:**

While baseline ALI was similar across cognitive statuses, longitudinal changes in specific neuroendocrine and inflammatory biomarkers, particularly Cortisol, Prolactin, IL‐18, and TNF‐α, were strongly associated with those with cognitive decline and AD progression. Future studies should identify clusters of AL biomarkers linked to cognitive decline across the AD continuum.

**Keywords**: Alzheimer's Disease, Mild Cognitive Impairment, Allostatic Load, Inflammatory Biomarkers, Neuroendocrine Biomarkers.

